# Using Immersive Virtual Reality to Enhance Social Interaction Among Older Adults: A Cross-Site Investigation

**DOI:** 10.1093/geroni/igad031

**Published:** 2023-04-13

**Authors:** Saleh Kalantari, Tong Bill Xu, Armin Mostafavi, Benjamin Kim, Andrew Dilanchian, Angella Lee, Walter R Boot, Sara J Czaja

**Affiliations:** Human Centered Design, Cornell University, Ithaca, New York, USA; Human Centered Design, Cornell University, Ithaca, New York, USA; Human Centered Design, Cornell University, Ithaca, New York, USA; Division of Geriatrics and Palliative Medicine, Center on Aging and Behavioral Research, Weill Cornell Medicine, New York City, New York, USA; Department of Psychology, Florida State University, Tallahassee, Florida, USA; Human Centered Design, Cornell University, Ithaca, New York, USA; Department of Psychology, Florida State University, Tallahassee, Florida, USA; Division of Geriatrics and Palliative Medicine, Center on Aging and Behavioral Research, Weill Cornell Medicine, New York City, New York, USA

**Keywords:** Older adults, Presence, Social engagement, Social VR, Usability

## Abstract

**Background and Objectives:**

Virtual reality (VR) applications are increasingly being targeted toward older adults as a means to maintain physical and cognitive skills and to connect with others, especially during the coronavirus disease 2019 era. Our knowledge about how older adults interact with VR is limited, however, since this is an emerging area and the related research literature is still rather slim. The current study focused specifically on older adults’ reactions to a social-VR environment, examining participant’s views about the possibility of meaningful interactions in this format, the impacts of social-VR immersion on mood and attitude, and features of the VR environment that affected these outcomes.

**Research Design and Methods:**

The researchers designed a novel social-VR environment with features intended to prompt conversation and collaborative problem-solving among older adults. Participants were recruited from 3 diverse geographic locations (Tallahassee, FL; Ithaca, NY; and New York City, NY), and were randomly assigned to a partner from one of the other sites for social-VR interaction. The sample consisted of 36 individuals aged 60 and older.

**Results:**

Reactions to the social VR were quite positive. Older adults reported high levels of engagement in the environment and perceived the social VR to be enjoyable and usable. Perceived spatial presence was found to be a central driver of positive outcomes. A majority of the participants indicated a willingness to reconnect with their VR partner in the future. The data also identified important areas for improvement that were of concern to older adults, such as the use of more realistic avatars, larger controllers more suited to aging hands, and more time for training/familiarization.

**Discussion and Implications:**

Overall, these findings suggest that VR can be an effective format for social engagement among older adults.

**Translational Significance:** Coronavirus disease 2019 has led to an expanding interest in electronic communication among older adults, and virtual reality (VR) technology is an exciting and powerful means of making such social connections. However, current social-VR applications were generally not designed with older adults in mind, and there has been little research into how older adults might use such platforms, or their specific needs in relation to the technology. The current study evaluated older adults’ reactions to a social-VR platform to help understand features that affected its usability and impact. The findings can help designers improve social-VR technologies specifically tailored for older adults.

Many older adults experience social isolation, defined as having few social relationships or infrequent social contact with others (Wu, 2020). It is well-documented that social isolation can have adverse impacts on emotional, cognitive, and physical health (Cotterell et al., 2018; Malcolm et al., 2019; National Academies of Sciences and Medicine, 2020; Nicholson, 2012; Wu, 2020). In a systematic review of studies on social isolation and loneliness, Malcolm and colleagues (2019) found that older adults are one of the populations most at risk, in part due to declines in mental and physical abilities leading to social withdraw. In recent years, the impact of the coronavirus disease 2019 (COVID-19) pandemic has contributed to a further increase in rates of social isolation among older adults, with the World Health Organization reporting that in some countries up to one third of the older population had regular feelings of loneliness (World Health Organization, 2021).

Increasing concerns about social isolation among older adults have arrived at a time in which digital technologies are becoming ubiquitous in our daily lives and are transforming the ways that we interact with each other. While these technological changes have significantly affected all age groups, older adults may face unique challenges in adapting to new communication technologies. Part of the reason for this is purely physical—older adults may face cognitive or manual accessibility barriers that make it difficult to learn and use new devices (Czaja et al., 2013). Additionally, some older adults may have a suspicion or lack of interest in new digital technologies due to their unfamiliarity, which frequently plays out through concerns about privacy and security (Kontos et al., 2014). This can lead to a strong resistance to engaging in any form of digital social interaction. Such avoidance may be viable for some older adults who have other means of social connection, but in today’s world it does constrain them from many opportunities.

For example, video chat platforms like Skype and Zoom, and social media such as FaceBook, are increasingly used as a primary means through which geographically distanced family members and friends stay in touch with each other (Kontos et al., 2014; Vroman et al., 2015). Older adults who avoid such platforms may be cut off from these engagements, as well as from information resources that are increasingly going online (Cotten et al., 2013). Digital communication platforms, when carefully designed and implemented, have been shown to be an effective means of decreasing feelings of isolation among older adults and helping them feel connected to friends and family (Czaja et al., 2018; Sen et al., 2022). A study by Cotten and colleagues (2013) found that Internet use by seniors was associated with higher levels of social support and a greater likelihood of participating in social activities. Similarly, a study by Mitzner and colleagues (2019) found that older adults who used social networking sites reported higher levels of social connectedness and social support compared to those who did not use these sites.

The desire for social interaction has also been identified as a primary motivation among older adults who participate in online virtual experiences (Siriaraya & Ang, 2012; Siriaraya et al., 2012). Virtual reality (VR) encompasses a wide range of technologies that enable immersive, three-dimensional environments with a strong sense of presence or “being there” in the artificial space, which may help to address the lack of perceived social presence that many older adults report in relation to other types of digital communication (Dilanchian et al., 2021; Suh & Prophet, 2018). VR is also coming to play a significant role in the realm of health care and skills rehabilitation, for example, through computerized training to enhance cognitive skills in older adults (Hill et al., 2017; Latikka et al., 2021; Liu et al., 2019), which has a strong potential to be combined with social interactions. One of the tremendous advantages of such platforms is that they can respond to the specific capabilities of individual users and be customized to account for the varied preferences and proclivities of a diverse range of older adults (Bol et al., 2019; Brandt et al., 2020; Fakoya et al., 2020).

## Prior Research on VR Use Among Older Adults

Although VR in general is sometimes regarded as a youth-oriented technology, research has shown that it is readily embraced by many older individuals, and that it has the potential to improve older adults’ sense of engagement and well-being (D’Cunha et al., 2019; Dermody et al., 2020; Huygelier et al., 2019; Lee et al., 2019; Thach et al., 2020). A handful of prior studies have been conducted to evaluate the use of VR by older adults with cognitive decline, and have generally found positive responses, feelings of engagement, and comfort with the technology when it was carefully introduced to participants (Appel et al., 2020; Arlati et al., 2021; Dilanchian et al., 2021; Kalantari et al., 2022; Park et al., 2020). It is important to note that responses in these studies varied quite a bit among different individual participants, and thus VR may not be suitable for all older adults. On average, however, there is a strong body of work supporting the use of VR as an engagement and cognition-enhancing mechanism for this population.

Beyond the use of VR as a cognitive-engagement environment, the topic of *social interactions* in VR poses an additional set of issues. The emergence of social-VR platforms can be traced back to long-standing aspirations toward collaborative spaces that erase geographic distance (Benford et al., 2001). Studies have found that interactions in VR spaces are perceived as being more “realistic” or more similar to in-person interactions when compared against other types of digital communications (Li et al., 2019). However, the ways in which people can use this relatively novel technology to socialize are not yet fully understood. The increasing popularity of social VR has led to an emerging research agenda focused on evaluating aspects of the mediated interactions that take place on such platforms. This includes studying design strategies for virtual social spaces (Jonas et al., 2019; McVeigh-Schultz et al., 2019; Sra et al., 2018; Williamson et al., 2021), communication modes and interactive activities in VR (Baker et al., 2019; Freeman & Maloney, 2021; Maloney & Freeman, 2020; McVeigh-Schultz et al., 2018; Moustafa & Steed, 2018), engagement strategies for long-distance couples and families (Maloney et al., 2020; Zamanifard & Freeman, 2019), and the psychology of VR self-presentation and avatars (Blackwell et al., 2019; Freeman et al., 2020; Freeman & Maloney, 2021; Kolesnichenko et al., 2019; Nowak & Fox, 2018). However, these studies have skewed strongly toward younger adults, and the specific study of older adults’ interactions with social VR has been extremely limited.

For older adults, design factors and intuitive interfaces may be particularly important in promoting the adoption of VR technology and comfort with using it (Roberts et al., 2019; Siriaraya & Ang, 2019; Syed-Abdul et al., 2019). A topic that has repeatedly emerged in the research literature is the design of avatars and full-body tracking to reflect real-world experiences (Darfler et al., 2022; Kalantari & Neo, 2020). Older adults tend to prefer more “realistic engagement” scenarios in VR that reflect ordinary environments and people, rather than “gamified” scenarios that bestow users with superhuman abilities to perform challenging tasks (Freeman & Maloney, 2021; Freeman et al., 2020). In one notable study, Baker and colleagues (2019) conducted workshops with older adults and found that the key drivers of VR technology’s acceptance in this population were *behavioral anthropomorphism* (“the embodied avatars’ ability to speak, move, and act in a human-like manner”) and *translational factors* (“how VR technology translates the movements of the aging body into the virtual environment”). Thus, the careful design of these environments is likely to be crucial to meeting the specific engagement needs of older populations, which may diverge from broader gamified development trajectories in the industry. For the potential of social VR to be realized among older adults it is imperative to collect additional information about how factors such as embodiment, presence, and engagement are experienced by older users. To date, most studies on the older adult population and VR have focused on nonsocial environments (in which participants interact with the VR environment independently of other users). In contrast, most studies on social VR have focused on younger adults. The current study was designed to help fill this gap by evaluating the responses of older adults to a social-VR application and analyzing features of the experience that may influence adoption of the technology.

## Research Questions

The study paired older adult participants from different geographic locations who had no prior social connections with each other. The participants engaged in various VR activities intended to promote conversation and collaboration. The study design was based around seven research questions, which are organized into three main categories: social experience, affective response, and usability.

In the area of social experience, our first and primary research question was: Will the VR-mediated interactions produce meaningful social experiences among older adults? (*RQ1*). We combined qualitative and quantitative approaches to address this question, using measures of perceived social presence (Nowak & Biocca, 2003) and Likeliness to Reconnect with Partner (Boothby et al., 2018), as well as post-immersion interviews. Second, based on prior literature that has explored the use of VR for restoration and relaxation among older adults (e.g., Appel et al., 2020; Huygelier et al., 2019; Kalantari et al., 2022), we also asked: Will the social-VR experience enhance the participants’ mood states? (*RQ2*). This was measured using the *Multidimensional Mood State Questionnaire* (Steyer et al., 1997). Finally, we wanted to evaluate if older adult individuals who engaged in pairwise social activities would demonstrate similar responses to the VR environment as their partners (*RQ3*). This was evaluated qualitatively through three variables: perceived social presence, Likeliness to Reconnect, and Engagement with the Environment. (A more detailed discussion of all measures used in the study is presented in Supplementary Material.)

In the area of affective responses, we sought to determine how such reactions to the VR activities were correlated with broader mood states and social outcomes (*RQ4*). This is important because understanding the impact of affective responses can help us determine what type of reactions to the content contribute to positive outcomes, thereby enhancing social-VR design approaches. The affective responses were measured using the *Self-Assessment Manikin (SAM)* developed by Bradley and Lang (1994), which is divided into measures of participants’ pleasure, arousal, and dominance (feeling of being in control). These factors were evaluated in relation to the Multidimensional Mood State Questionnaire and to measures of perceived social presence and Likeliness to Reconnect.

The third category of research questions focused on technology acceptance and usability. Prior work has suggested that even brief exposure to VR may improve older adults’ attitudes toward the technology (Appel et al., 2020; Huygelier et al., 2019; Kalantari et al., 2022). We sought to determine if this would hold true for our social-VR program, using a scale developed by Huygelier and colleagues (2019) for the specific purpose of evaluating the *Acceptance of Head-mounted Virtual Reality in Older Adults* (*RQ5*). In addition, we looked for the possibility of negative impacts in the older adult population, including motion sickness (as per Kennedy et al., 1993) or excessive cognitive workload (as per Hart & Staveland, 1988), and we measured the participants’ ratings of the technology on Finstad’s (2010)*Usability Metric* (*RQ6*). Based on previous studies suggesting links between perceptions of spatial presence and engagement in VR (Barreda-Ángeles & Hartmann, 2022; McCreery et al., 2015), we also sought to evaluate if perceived spatial presence in the VR (measured using the *MEC Spatial Questionnaire*; Vorderer et al., 2004) would predict the social outcomes in terms of perceived social presence and Likeliness to Reconnect (*RQ7*).

## Method

In designing the content of our social-VR program, we accounted for the prior research literature (as discussed in the previous section) related to movement affordances, proxemic spacing, avatar customization, gesture and posture control for avatars, and social activity design. The engagement tasks we incorporated were based on McGrath’s circumplex model, which is split into four quadrants: generating a variety of ideas or plans, choosing a solution, negotiating with contradicting views, and executing the revised solution (McGrath, 1984). We also used a participatory approach during the design process by directly engaging with older adults to give us feedback about the planned VR components. This involved inviting older adults to our lab (using a convenience recruiting approach) and asking them to test various interaction scenarios, which were then fine-tuned based on the feedback.

**Figure 1.**
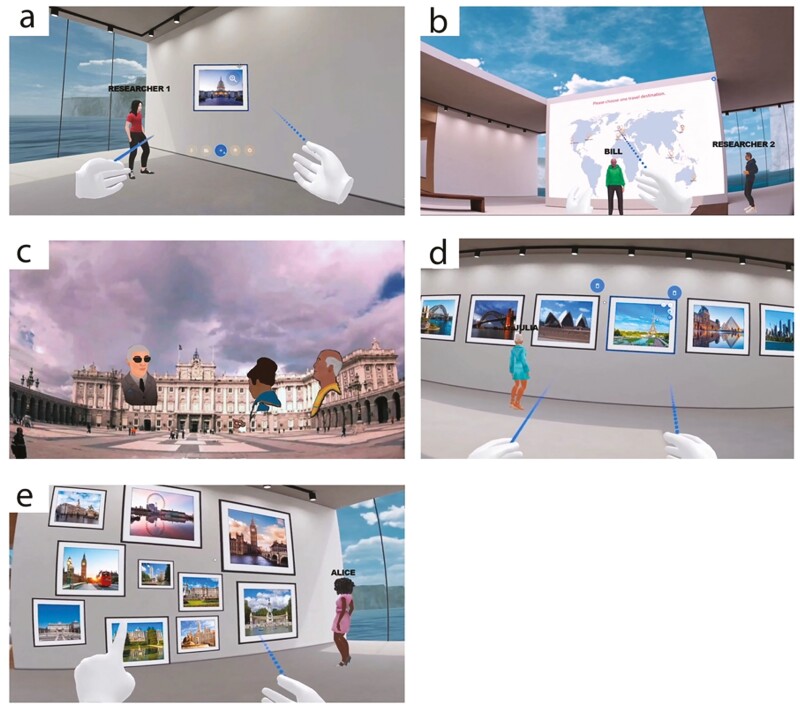
Screenshots from the participants’ view in the virtual reality modules: (A) Training module; (B) Introductions module; (C) Travel module; and (D and E) Productive Engagement module.

The final social-VR product for this study included four modules, which we labeled as Training, Introductions, Travel, and Productive Engagement. The centerpieces of the experience were the “Travel” module, in which participants viewed 360° immersive videos of international destinations that they selected, and the “Productive Engagement” module, in which they solved memory puzzles and engaged in creativity tasks. Screenshots from the VR experience are shown in Figure 1. A more detailed description of the module contents and their technological development is presented in Supplementary Material.

### Participants

The evaluation of the social-VR program was designed as a multisite study, encompassing participants in Ithaca, NY, New York City, NY, and Tallahassee, FL. We recruited 12 participants at each site (36 total) using a convenience sampling method. The researchers contacted each individual participant by phone to discuss the study activities, exclusion criteria (epilepsy, motion sickness, medical implants), time commitment, and scheduling. The participants were then sent an informed-consent document and a demographic questionnaire, which they were asked to complete prior to the experiment session. Once everyone’s availability was obtained, the researchers paired each participant with a partner from a different city and scheduled a joint session, in which each of the partners would visit the lab sites in their respective cities to use the VR equipment. The final data set included six pairings between New York City and Ithaca, six pairings between New York City and Tallahassee, and six pairings between Ithaca and Tallahassee.

**Figure 2.**
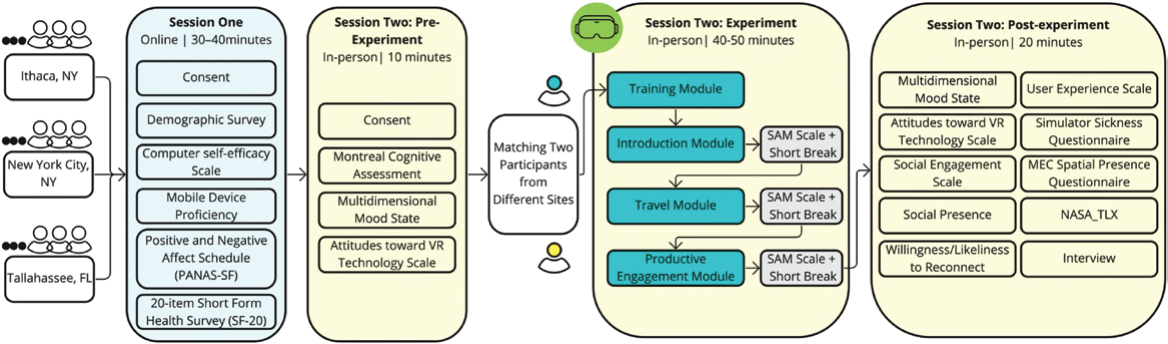
Summary of measurement tools used in the study and the study flow. SAM = Self-Assessment Manikin; VR = virtual reality; NASA_TLX = NASA Task Load Index.

We asked participants to complete a variety of demographic instruments, including the *Computer Self-efficacy Scale* (Barbeite & Weiss, 2004), a *Short Form Health Survey* (Cooke et al., 1996), and the *Montreal Cognitive Assessment* (MoCA; Nasreddine et al., 2005), among others. All instruments are described in detail in Supplementary Material; due to space constraints they can only be listed briefly here. A summary overview of the instruments and administration sequence is presented in Figure 2, and descriptive statistics are presented in Tables 1 and 2. All participants were over 60 years of age, with an average age of 71 (standard deviation [*SD*] = 5.2). The overall sample skewed female (72%) and White (81%).

**Table 1.** Participant Characteristics

Variable	Ithaca (*n* = 12)	New York City (*n* = 12)	Tallahassee (*n* = 12)	Overall (*N* = 36)
Age in years, *M* (*SD*)	66.8 (4.0)	71.3 (4.8)	74.3 (4.3)	70.8 (5.2)
Gender				
Female	7 (58%)	11 (92%)	8 (67%)	26 (72%)
Male	5 (42%)	0 (0%)	4 (33%)	9 (25%)
Nonbinary/third gender	0 (0%)	1 (8%)	0 (0%)	1 (3%)
Ethnicity, *n* (%)				
Other	3 (25%)	1 (8%)	0 (0%)	4 (11%)
White	9 (75%)	8 (67%)	12 (100%)	29 (81%)
Black or African American	0 (0%)	3 (25%)	0 (0%)	3 (8%)
Education, *n* (%)				
4-year degree	5 (42%)	3 (25%)	2 (17%)	10 (28%)
Doctorate	1 (8%)	0 (0%)	1 (8%)	2 (6%)
Professional degree	5 (42%)	8 (67%)	6 (50%)	19 (53%)
Some college	1 (8%)	1 (8%)	3 (25%)	5 (14%)
MoCA, *M* (*SD*)	27.3 (1.8)	25.7 (3.7)	27.3 (1.5)	26.8 (2.6)
Computer proficiency, *M* (*SD*)	27.7 (2.1)	26.3 (3.6)	27.7 (3.0)	27.2 (2.9)
Mobile proficiency, *M* (*SD*)	33.3 (6.4)	33.4 (5.1)	33.7 (8.5)	33.5 (6.6)
PANAS, *M* (*SD*)				
Positive affect	38.4 (6.3)	38.5 (7.1)	34.2 (6.0)	37.0 (6.6)
Negative affect	11.8 (3.3)	14.9 (5.6)	13.1 (3.8)	13.3 (4.4)
SF-20, *M* (*SD*)				
Physical functioning	84.0 (24.7)	75.0 (30.4)	75.0 (27.1)	78.0 (27.0)
Role functioning	91.7 (28.9)	79.2 (33.4)	77.1 (39.1)	82.6 (33.7)
Social functioning	88.3 (21.7)	81.7 (31.3)	83.3 (31.7)	84.4 (27.9)
Mental health	76.3 (21.1)	75.0 (13.4)	77.3 (13.6)	76.2 (16.0)
Health perceptions	71.1 (28.8)	64.9 (29.2)	69.8 (30.8)	68.6 (28.9)
Pain	33.3 (26.1)	35.0 (28.4)	38.3 (28.9)	35.6 (27.1)

*Notes*: MoCA = Montreal Cognitive Assessment; PANAS = Positive and Negative Affect Schedule; *SD* = standard deviation; SF-20 = 20-Item Short Form Health Survey; *M* = mean scores.

**Table 2.** Descriptive Statistics for All Outcome Variables

Outcome variable	Mean (*SD*)	Theoretical range
Usability	67.0 (20.7)	[0, 100]
Motion sickness	21.8 (26.7)	[0, 235.62]
Engagement	4.2 (0.9)	[1, 5]
Spatial Presence		
Self-location	3.8 (1.3)	[1, 5]
Possible actions	3.7 (1.2)	[1, 5]
Social Presence	61.2 (22.0)	[0, 100]
Workload	2.8 (1.2)	[1, 7]
Reconnect	3.7 (0.8)	[1, 5]
Attitude		
Pre	3.2 (0.4)	[1, 5]
Post	3.1 (0.3)	[1, 5]
Change	−0.1 (0.4)	[−4, 4]
Mood		
Good–bad		
Pre	48.3 (7.4)	[10, 60]
Post	49.8 (8.5)	[10, 60]
Change	1.5 (6.3)	[−50, 50]
Awake–tired		
Pre	47.0 (6.9)	[10, 60]
Post	47.6 (7.6)	[10, 60]
Change	0.6 (6.6)	[−50, 50]
Calm–nervous		
Pre	44.3 (7.9)	[10, 60]
Post	47.3 (8.1)	[10, 60]
Change	3.0 (6.9)	[−50, 50]
Affect scores		
Pleasure	2.3 (1.4)	[1, 5]
Arousal	4.7 (2.1)	[1, 5]
Dominance	5.1 (1.5)	[1, 5]

Note: *SD* = standard deviation.

### Study Procedures

All study procedures and questionnaires were approved by the Institutional Review Boards at all three institutions, including Cornell University, Weill Cornell Medicine, and Florida State University prior to the research activities. During the experiment sessions there were two researchers present at each site: one was responsible for administering the questionnaires and monitoring safety issues, and the other was responsible for coordinating the technological setup and served as a moderator within the virtual environment. All three study sites used a consumer version of the Oculus Quest 2 head-mounted display and Oculus handheld controllers. The headsets were customized with the Oculus Elite Strap (an adjustable ergonomic support). All sites used a Blue Yeti USB microphone and a Sony SRS-RA3000 speaker for transmitting sound through Zoom. We recorded the VR display video of participants using the Side Quest app (www.sidequestvr.com).

The participants filled out most of the demographic questionnaires online before arriving at the study sites. When participants arrived for their sessions the researchers administered the MoCA and questionnaires assessing mood states and attitudes toward VR. During this time, photos of the participants were taken and used to create virtual avatars. The researchers then helped each participant to don the VR equipment and prepare to enter the modules. Participants sat in a swivel chair or stood as desired throughout the experiment activities, and they could move around in a space that was approximately 2 m × 2 m. A 5-min break was given after each module, with participants’ voice communication temporarily muted between the study sites. The start of each module was synchronized in real time by the researchers, so that one participant would not have to wait a significant time in the VR environment for the other participant to arrive.

Completing the Training and Introductions modules took around 5–10 min total. Once the Travel module began, the moderator/researcher began to gradually withdraw from involvement in the VR, encouraging the participants to engage in organic dialogue about whatever topics they wished, and to support each other with questions or issues with the controllers. For the rest of the VR session the researcher would only provide prompts if participants directly asked for their assistance or if both participants remained continuously silent for more than 2 min. Completing the Travel module took around 12–15 min (the researchers ended the videos during an organic lull in the conversation) and completing the Productive Engagement module took around 15–20 min (ending when the participants expressed satisfaction with their photo collage design). After the Introductions, Travel, and Productive Engagement modules, participants completed the Self-Assessment Scale (SAM) to measure affective responses to the VR activities. After the VR activities were finished, each participant completed several additional questionnaires, followed by a semistructured interview about their experiences. Each participant who completed the experiment received a $50 gift card as compensation for their time.

## Results

We used the R programming language for statistical data analysis. In addition to conventional descriptive results, we also calculated the intracluster correlation coefficient (ρ) using the library “fishmethods,” following Lohr’s equation no. 5.8 (Lohr, 2021, p. 139). To better handle the variance within each experimental pair, we fitted linear mixed models with the library “lme4,” then estimated and compared marginal means with Satterthwaite approximation using the library “emmeans.” For analyzing changes in mood and attitudes toward VR, we fitted linear mixed models with no fixed effect and random effects of participant pairs to estimate the change in dimensions of mood before and after the experiment and compared them versus a baseline of zero change. As an exploratory analysis, we also fitted linear mixed models with fixed effects of likelihood of mild cognitive impairment (CI; based on a MoCA screening score with a threshold of 26) and random effects of participant pairs to estimate the differences between CI and non-CI groups. For testing the correlation between participants’ affective responses to the VR activities with their mood states and with social outcomes, we fitted linear mixed models with fixed effects for the affective responses (happy–unhappy, excited–bored, and controlled–dominant dimensions of the SAM), and random effects of participant pairs, to estimate the social outcomes and engagement. To evaluate the impact of spatial presence as well as mediating variables, we used a structural equation modeling approach (Hooper et al., 2008), using the R library “lavaan.” Note that these results do need to be interpreted with caution due to the small sample size (*n* = 36).

**Figure 3.**
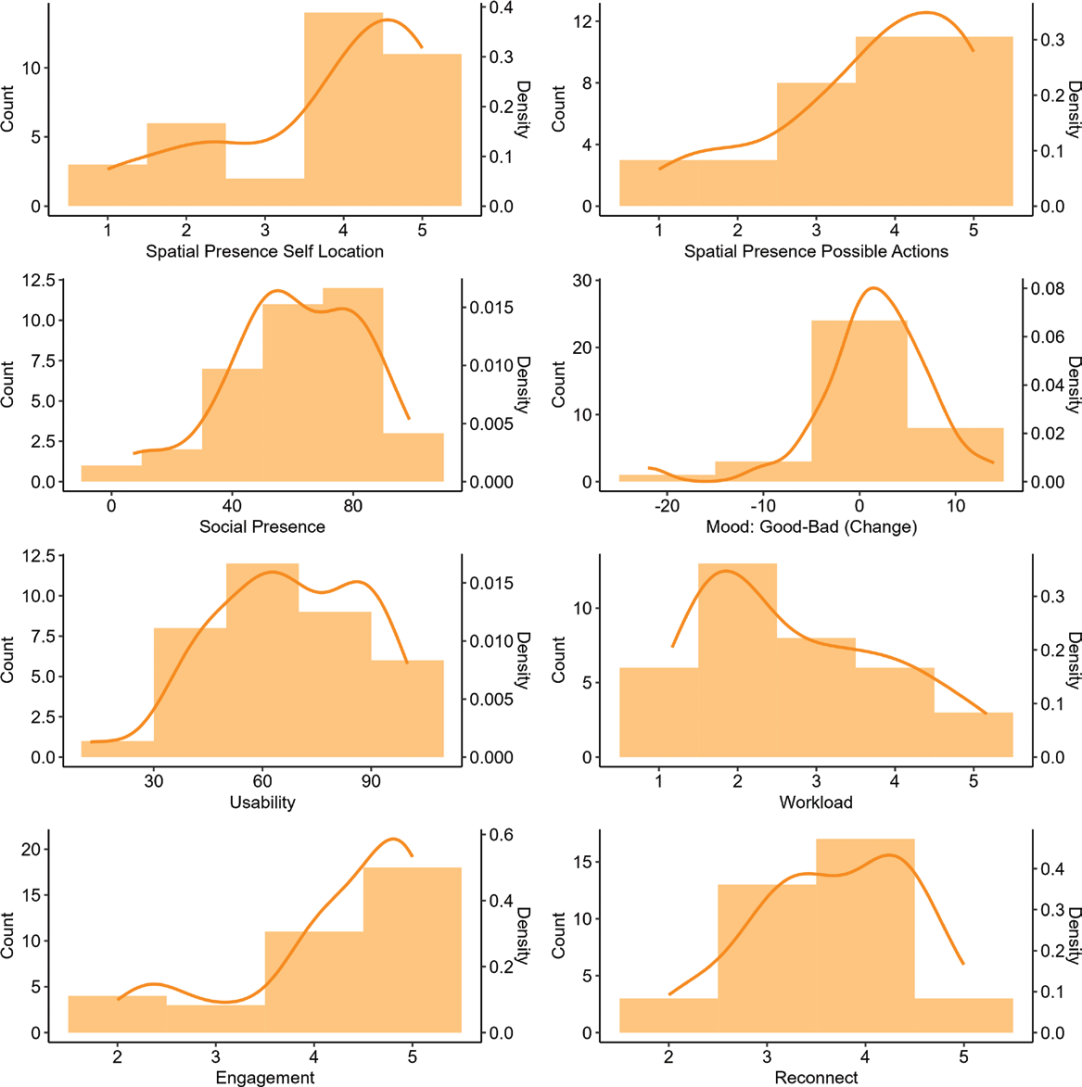
Distribution of outcome measures.

Participants reported a very high level of overall Engagement in the VR environment (*M* = 4.18 out of 5.00; *SD* = 0.91). A summary of the descriptive statistics for all outcome variables is presented in Table 2 and Figure 3. More information about the descriptive results for outcome variables evaluating different aspects of the social interactions, including specific questions on the Engagement, Social Presence, and Reconnect scales, and descriptive results for different affective response (SAM subscales), are reported in Supplementary Tables S1–S4.

### Participant Experiences in the Social-VR Intervention (RQ1 and RQ2)

**Figure 4.**
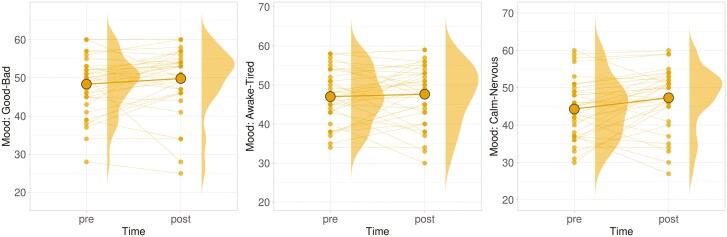
Change in mood from before to after the virtual reality experience (statistically significant differences were found only for the calm–nervous dimension, at right).

The participants scored moderately high on ratings of perceived social presence in the VR environment (*M* = 61.21 out of 100; *SD* = 22.00), and on ratings for Likeness to Reconnect with the VR partner (*M* = 3.69 out of 5; *SD* = 0.79). In terms of their Mood States, changes in mood states were small but positive, with an average 1.47-point shift toward the “good” mood state (*SD* = 6.30), an average 3.00-point shift toward the “calm” mood state (*SD* = 6.93), and an average 0.61-point shift toward the “awake” mood state (*SD* = 6.61), all measured out of a theoretical range of [−60 to +60]. Only the change in the calm–nervous dimension of mood was found to be significant (*t*(17.0) = 2.53; *p* = .022; 95% confidence interval [CI]: [0.61, 5.39]). This finding makes intuitive sense, as participants would be expected to feel more nervous at the beginning of an experiment session, and it therefore may not be related to the features of the VR experience (Figure 4). Interview findings are reported further below.

### Similarity in Reconnect, Social Presence, and Engagement Within Pairs (RQ3)

We found small intracluster correlations for both Likelihood to Reconnect and Social Presence within the participant pairs (correlation coefficient ρ = 0.08 and 0.11, respectively). This indicates that the effect of pairing had a small but statistically significant impact on these two variables. There was a somewhat larger intracluster correlation for Engagement with the VR environment (ρ = 0.36). These findings were expected, as positive social relationships are generally bidirectional, and the engagement behaviors of a partner in VR are likely to affect one’s own sense of engagement and interest in the program’s content.

### The Impact of Affect on Mood Changes, Social Outcomes, and Engagement (RQ4)

Some affective reactions to the virtual experience as measured by the SAM instrument were predictive of mood changes. The pleasure (happy–unhappy) dimension of the SAM was a predictor of changes in all measured mood states, including good–bad mood (*b* = −1.45; standard error [*SE*] = 0.71; *t*(32.0) = −2.05; *p* = .049), awake–tired mood (*b* = −1.92; *SE* = 0.72; *t*(32.0) = −2.67; *p* = .012), and calm–nervous mood (*b* = −2.01; *SE* = 0.80; *t*(32.0) = −2.52; *p* = .018). The arousal (excited–bored) dimension was a marginal predictor of changes in good–bad mood (*b* = −0.91; *SE* = 0.50; *t*(32.0) = −1.85; *p* = .075) and changes in awake–tired mood (*b* = −0.86; *SE* = 0.50; *t*(32.0) = −1.71; *p* = .098). The dominance dimension (in control/not in control) was not a significant predictor of changes in any of the mood dimensions.

The pleasure dimension of the SAM was also found to be a predictor of Likeliness to Reconnect (*b* = −0.21; *SE* = 0.09; *t*(32.0) = −2.33; *p* = .027) and Engagement with the VR Environment (*b* = −0.22; *SE* = 0.10; *t*(27.25) = −2.18; *p* = .039). The arousal dimension of the SAM was a marginal predictor of Engagement (*b* = −0.13; *SE* = 0.07; *t*(25.09) = −1.92; *p* = .068). The dominance dimension of the SAM was not a significant predictor of Likeliness to Reconnect or Engagement.

### Participant Attitudes and Usability of the Social-VR Platform (RQ5 and RQ6)

There was only a small change in participants’ attitudes toward VR technology from before the experimental session to after the session (*M* = −0.05 out of [−4 to +4]; *SD* = 0.37), the change is not significant, *t*(35.0) = −0.849, *p* = .402.

The participants reported that the VR system had a high usability (*M* = 67.01 out of 100; *SD* = 20.73). There was some simulator sickness reported, but the rates were low (*M* = 21.82 out of 235.62; *SD* = 26.69). Task workload scores were also within the low range (*M* = 2.86 out of 7; *SD* = 1.17), indicating that the VR experience was not very mentally taxing for the participants.

### Exploratory Analysis of CI

**Figure 5.**
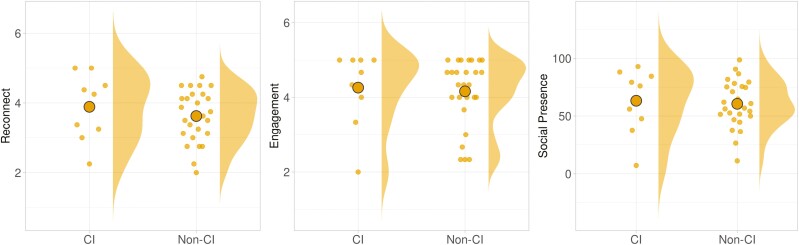
Impacts of cognitive impairment for social variables (statistically significant differences were found only for Likelihood to Reconnect, at left).

**Figure 6.**
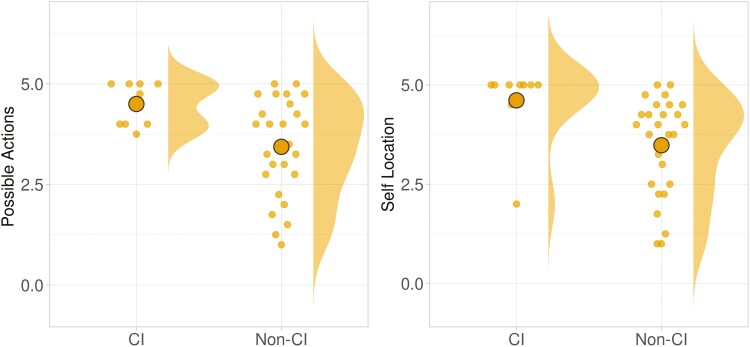
Impacts of cognitive impairment (CI) for spatial presence (statistically significant differences were found for both the “Self-Location” and “Possible Actions” dimensions).

We found a relatively large portion (25%) of the participants had MoCA scores that would qualify them as likely to be experiencing mild CI. (The MoCA is merely a screening instrument, and the researchers are not health care professionals, so this does not constitute a diagnosis; nonetheless, it was an interesting aspect of our sample.) We decided post hoc to conduct an exploratory analysis of the effect of CI on other study variables. Most variables did not yield any significant findings in this regard, but the results indicated significant differences in the two spatial presence subscales: Possible Actions (Δ = 1.07; *SE* = 0.41; *t*(32.8) = 2.60; *p* = .014; 95% CI: [0.23, 1.90]) and Self-Location (Δ = 1.17; *SE* = 0.43; *t*(30.7) = 2.72; *p* = .011; 95% CI: [0.29, 2.05]). Participants with likely CI tended to have a stronger sense of spatial presence (“being there”) within the VR. The participants with likely CI also tended to report a higher Likeliness to Reconnect with partners (Figures 5 and 6). These findings should be considered highly provisional because the sample size for CI participants was quite small (*n* = 9; 25%).

### Spatial Presence and Other Factors Predicting Social Outcomes and Engagement (RQ7)

Overall, the participants reported a moderately high sense of spatial presence in the VR, on both subscales of Possible Actions (*M* = 3.70 out of 5; *SD* = 1.17) and Self-Location (*M* = 3.76 out of 5; *SD* = 1.27). We evaluated correlations between spatial presence and the other primary study variables to construct a structural equation model. Table 3 presents the correlation coefficients between variables in the model. Notably, we combined the two subscales of spatial presence in this analysis (by adding them together) due to a very high level of correlation between those subscales (*r* = 0.86, *p* < .001). We also allowed the residuals of Reconnect and Usability to correlate, under the assumption that participants might be thinking about reconnecting via the same platform when reporting Likeliness to Reconnect.

**Table 3.** Correlations Between Variables Used for the Structural Equation Model

Variable	Reconnect	Engagement	Social Presence	Mood	Spatial Presence	Usability
Engagement	0.79*** (<.001)					
Social Presence	0.19 (.941)	0.35 (.330)				
Mood (good–bad) change	0.41 (.176)	0.53* (.018)	0.15 (.941)			
Spatial Presence (all)	0.43 (.132)	0.57** (.005)	0.41 (.176)	0.46 (.088)		
Usability	0.23 (.941)	0.45 (.093)	0.23 (.941)	0.24 (.941)	0.36 (.330)	
Self-Assessment Scale (happy–unhappy)	−0.36 (.330)	−0.38 (.263)	−0.13 (.941)	−0.35 (.330)	−0.28 (.711)	−0.55** (.010)

*Notes*: *r* (*p* value after Holm correction). Spatial presence here is an additive combination of the “Possible Actions” and “Self-Location” subscales of the MEC, and mood (good–bad) is the mood change before and after the experiment.

*p < .05. **p < .01. ***p < .005.

**Figure 7.**
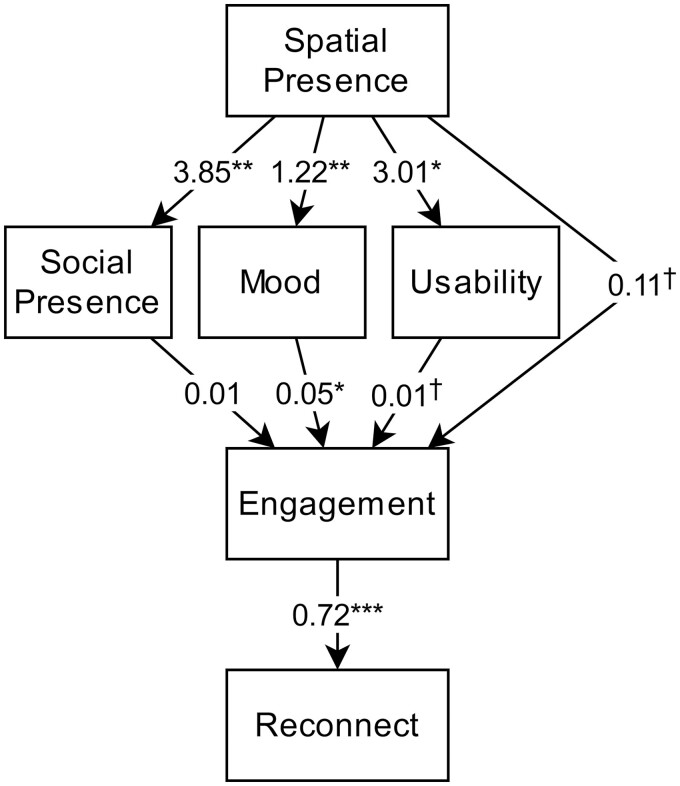
Structural equation model for the main study variables. ^†^*p* < .1; *p < .05; **p < .01; ***p < .005.

The model that we derived is shown in Figure 7. Most of the paths, except the one from social presence to engagement, were found to be significant or marginally significant. With maximum likelihood estimator, the model has a chi-square of 1.884 (6, *N* = 36); *p* = .930; GFI (Goodness of Fit) = 0.982; AGFI (Adjusted Goodness of Fit) = 0.937; CFI (Comparative Fit Index) = 1.000; RMSEA (Root Mean Square Error of Approximation) < 0.001, 90% CI (Confidence Interval): [0.000, 0.063], *p*[<.05] = .943; SRMR (Standardized Root Mean Square Residual) = 0.039, indicating a relatively good fit. However, sample size issues are an important concern when constructing structural equation models, and the rule-of-thumb is often regarded as 10 cases per parameter (Wolf et al., 2013), which our study does not meet. Therefore, we have applied Swain’s (1975) correction in evaluating our model, which has been shown to account for bias caused by limited sample size with an *N*:*t* ratio (case per parameter) greater than 2:1 (Herzog & Boomsma, 2009; Herzog et al., 2007). The adjusted fit measures, which account for the small sample size, still indicated that the model was a reasonably good fit (chi-sq = 1.754 (6, *N* = 36), *p* = .940; CFI = 1; RMSEA < 0.001, 90% CI: [0.000, 0.064], *p*[<.05] = .951).

### Qualitative Interview Analysis

We used a qualitative content-analysis approach (Elo & Kyngäs, 2008) to parse the participants’ responses from the exit interviews. The purpose of these interviews was to obtain more detailed feedback from the participants about their social-VR experience, including its overall ability to produce meaningful social connection and aspects of the technology and content design that affected the experience. The exit interview consisted of the following four questions:

1. How would you describe your VR social interaction experience to a friend or family member?
2. Which part did you like most and why?
3. Which part could be improved and why?
4. Would you use this tool for social interaction with people you know? Would you use it with people you don’t know?

All responses were transcribed by a research assistant, who also developed a preliminarily coding schema based on commonly voiced themes related to the research variables. Two other research assistants independently reviewed the transcripts and coded the data segments by theme. After the research assistants completed their independent coding, they met to collaboratively resolve any disagreements about how the text segments were organized. The resulting themes and some example quotations are presented in Table 4.

**Table 4.** Qualitative Content Analysis of Exit Interview Responses

Theme	Subtheme level 1	Subtheme level 2	Number of responses	Example quotes
Affective response	Positive		81	“[VR] was a fun and exciting experience.” (D013)
	Negative		10	“A bit disappointing.” (F008)
Mood change	Bad mood to good mood		8	“Once I finally got the hang of it a little bit. Initially a bit awkward by clicking the wrong button and other things that were harder. Once I became more competent in it, I was able to do more, so it was less intimidating over time. When it was intimidating it was not so fun.” (W013)
	Good mood to bad mood		0	
Likeliness to Reconnect	Likely	With familiar people	26	“I would definitely use it for people that I do know.” (D013)
		With strangers	23	“Yes I would use this tool for social interaction with other people, whether I know them or not.” (W024)
	Unlikely	With familiar people	10	“My daughter is in Philadelphia; that was who called me. So if she … I would rather be with her than in VR.” (D002)
		With strangers	9	“For those I don’t know … not sure if I would trust people I don’t know to use this [VR] with.” (W003)
Social Presence	Present		25	“Well I would point out that if you were in a [real-world] location with another person where your conversation was no different.… It’s just that the environment is created virtually in such a way as you feel that you are actually immersed in it and the communication is no different.” (D008)
	Nonpresent		24	“If this is a study on how people develop friendships or socialization in virtual reality, then I don’t understand how this would happen.” (W028)
Spatial Presence	Present		28	“I like the idea that it’s so three-dimensional that you really do feel like you are in a different place. Even though you realize it’s imaginary it somehow feels realistic on a certain level so I like that part of it a lot.” (D010)
	Nonpresent		12	“I first felt limited by not seeing my own avatar, therefore I didn’t see myself, and I do see myself in reality (my arms and legs). I also found walking to the next location rather strange.” (W003)
Usability	Usable		23	“The ease of movement and the ease of moving objects.” (D002)
	Unusable		39	“The headset needs to be more accommodating to an older person. Being able to work with glasses, and not being quite so heavy.” (W013)

*Note*: VR = virtual reality.

One notable aspect of this data is that affective responses to the VR environment were predominantly positive (81 positive statements vs 10 negative statements). Terms such as “comfortable,” “fun,” and “interesting” were common in the interviews. Such statements were most frequently linked to the 360° videos in the Travel module. A small number of participants expressed a negative reaction to the VR activities, mostly using terms that conveyed a sense of being overwhelmed, such as “confusing” and “nerve-racking.” The presence of such negative experiences among the participants supports the view that VR may be experienced in very different ways by different individuals in the older adult population. It is also notable that responses indicating mood changes were all in the direction of worse mood in the beginning of the VR sessions to better mood at the end of the sessions. These positive changes in mood were commonly linked to feelings of increasing success or mastery in the VR.

Social presence and nonpresence were frequent themes. For example, one participant (D008), a 73-year-old, male Ithaca resident, characterized the VR environment as just another place to carry out desired social activities: “the communication is no different [in the VR].” This participant’s conversation partner (F014), a 74-year-old, female Tallahassee resident, shared a similar sentiment: “I would love to have access to use this [VR] to interact with others.” In this case, both participants appeared to regard the VR as a “transparent” medium that did not interfere with reaching their social goals. Such participant pairing effects were notable in the exit interview data, as the researchers frequently found similar responses (positive or negative) expressed by both partners in a VR session. On the negative side, several participants mentioned the inability to clearly read body language and gauge a partner’s emotions as important issues. However, overall, the interview data indicated a fairly strong interest in using the VR platform again, especially if there was a lack of opportunities to visit someone in the physical world. Interest in using the platform to connect with new individuals (strangers) and with familiar individuals (friends and family) was expressed with approximately the same frequency.

Participants also mentioned the feeling of spatial presence as an important aspect of their VR interactions. One participant (D001), a 67-year-old, female Ithaca resident with likely CI, indicated a feeling of immersion in the 360° videos that extended to curiosity about the individuals captured in those videos: “I felt I was eavesdropping on the groups seen and people moving around and I find that very exciting, trying to imagine what their conversations were.” In this case, the participant considered “people watching” with her actual conversation partner as an enjoyable part of the experience. In contrast, several participants expressed frustration with not being able to move around voluntarily during the 360° video environments and said this reduced their sense of immersion. The inability to see one’s own avatar in our setup was also frequently mentioned as a technological obstacle to the feeling of spatial presence.

Although the usability ratings were high, participants provided specific recommendations to enhance the VR system. One valuable suggestion was to incorporate a survey-based partner-matching component, to help ensure that individuals would be paired with amiable partners who shared common interests. Several participants indicated that they felt there was not enough time allotted during the experiment to complete training at a measured pace, fully engage with partners, and explore the available tasks. Another recommendation was to use a wireless headset. Issues with the headset were commonly mentioned in the interviews, including complaints about its weight, a lack of compatibility with some eyeglasses, and feelings of awkwardness or discomfort. There were also multiple suggestions to improve the handheld controller by making it larger and less complex, thus reducing the required finger dexterity. These hardware accessibility issues are crucial considerations, and they indicate that the manufacturers of VR systems have not yet given sufficient consideration to the needs of older adults in their product designs.

Finally, some participants indicated that they would enjoy utilizing the VR technologies to emulate certain physical activities, especially ones that they were not able to currently perform in real life due to COVID-19 restrictions or mobility issues. Some of the activities mentioned by participants in this regard included tossing a ball with a partner, going bowling, and visiting a familiar physical location such as a local theater or restaurant.

## Discussion

Our central finding is that the majority of the older adult participants found the social-VR environment to be a positive experience, with high ratings for Engagement and for Willingness/Likelihood to Reconnect with a partner. These positive findings are congruent with and lend further support to the limited number of studies that have previously evaluated older adult’s responses to social VR (Baker et al., 2019; Roberts et al., 2019; Siriaraya & Ang, 2019). We found that social VR can be rewarding for older adults, but also that aspects of the system, ranging from the pacing of activities, to the extent of environmental realism, to the physical design of hand controllers and other equipment, may need adjustment to better account for the needs of this population. Our study also concurred with prior findings (Dermody et al., 2020; Huygelier et al., 2019; Kalantari et al., 2022) in showing relatively low levels of simulator sickness and task workload among older adults engaging with VR. It should be noted, however, that the short-term immersion sessions conducted our experiment do not predict long-term outcomes. The positive reactions expressed by participants may have been affected by the novelty of interacting with a new technology and taking part in a research study (Vaportzis et al., 2017), as well as a variety of other confounding variables, and these positive effects might not hold up through repeated daily use of VR. Further research is needed to evaluate the longitudinal impacts of persistent social-VR use by older adults.

While previous studies have found significant changes in mood states and in attitudes toward VR in various populations after brief exposure to the technology (Huygelier et al., 2019; Kalantari et al., 2022), we did not observe such effects, except for in the single calm–nervous dimension of the *Multidimensional Mood State Questionnaire* (which might be explained as an outcome of familiarization as nervousness associated with the new technology subsided). The reasons why we did not find broader changes in attitude and mood in our experiment are uncertain, but it may be related to our participants’ relatively high familiarity with information technology, as indicated in the education demographics and Computer Proficiency instruments (Table 1). Mood states and attitudes toward VR were already fairly high among the participants in the pre-experiment measures (Table 2), and thus there was less room for these measures to improve. Additionally, prior studies that found mood effects were focused not on social VR but rather on restorative environments such as virtual gardens that were oriented explicitly toward the goal of mood improvement.

Participants reported a moderately high level of perceived social presence in the VR (mean 61.21 out of 100). However, the variance on this measure was quite large (an *SD* of 22.00). This was also reflected in our interviews, in which some participants were very enthusiastic about the ability to make social connections in the VR, while other participants were skeptical and indicated that it did not feel like a “real” human interaction. There are important technological features that can contribute to feelings of social presence, most notably avatar realism. Even so, the wide range of responses in our study would seem to indicate that some older adults may simply be more amiable to the context of virtual socializing compared to others. More research should be conducted to evaluate if these differences are related to specific user characteristics, personality features, or social goals.

In an exploratory analysis of the impact of mild CI we did not find many significant correlations with the other study variables. The greater sense of spatial presence found among CI participants in our study, however, should be considered in future research and product development, as it may indicate a greater risk of such participants becoming “lost” in the simulation. Our findings in this regard are quite provisional, given the small number of CI participants, and we recommend that future researchers continue to look closely at this factor.

We also found that affective responses to the environment—particularly the degree of reported pleasure and excitement—were correlated with Engagement and with Likeliness to Reconnect. This implies that designers should strive to integrate functionalities that produce pleasure and excitement as a means of enhancing the social outcomes. Identifying appropriate content for this purpose is a complex task, as it entails creating balanced and refreshing experiences that remain “new” without being confusing or overwhelming. Fortunately, user testing of VR environments and subsequent refinements of the content are relatively easy to implement (compared to real-world designs).

Finally, the structural equation model that we developed to analyze mediation relationships indicated that spatial presence was a key factor for predicting Engagement and positive social experiences (as measured by Likeliness to Reconnect). Mood change and Usability were identified as the most important variables mediating the relationship between spatial presence and Engagement, and Engagement was the variable that had the most impact on Likeliness to Reconnect (Figure 6). This indicates that efforts to make the VR environment more immersive can have a tremendous impact on the response to a VR program and ultimately on the social outcomes. Numerous suggestions to help improve spatial presence emerged in our study, including better nuance, performance, and visibility of avatars (e.g., being able to see portions of one’s own avatar body when looking around), and hardware improvements to reduce physical distraction (e.g., lightweight and wireless headsets). It is interesting that nonsocial aspects of the VR (spatial presence, usability, aroused and positive mood) appear to be driving Engagement and through that driving social outcomes, rather than the other way around. This finding has overlap with previous studies (Barreda-Ángeles & Hartmann, 2022; Jonas et al., 2019; McCreery et al., 2015; Siriaraya & Ang, 2012) that identified the sense of spatial presence in VR as a central factor affecting the quality of social interaction.

### Limitations and Future Research Directions

The sample size in this pilot study was relatively small, which limits statistical power and our ability to detect relationships among the variables. This is particularly notable in regard to our comparison of participants with likely CI versus those without. Future research would benefit from expanded sample sizes, which would allow a better evaluation of other demographic variables that may intersect with participant age. Among the important participant characteristics that could potentially affect social-VR experiences are factors such as employment status, relationship status, introversion/extroversion, gender, prior experience with VR, and many other variables that were not evaluated in the current work. Longitudinal studies to evaluate the effects of social-VR programs over time are also strongly needed, as short-term exposure outcomes may not predictably translate into long-term effects.

The study was also limited by the VR program design. Some of the findings may not be fully generalizable to other VR engines, and the rapid pace of technological development in this fields means that innovations such as body tracking and facial tracking may soon have powerful impacts on VR experiences and outcomes (Baker et al., 2019). In regard to the content, designing and evaluating a variety of different VR social activities in future research will help to produce important data about types of experiences that are most valuable and of interest to older adult populations. A feeling of insufficient immersion time to become familiar with the system, to go through the training at a relaxed place, to engage fully with the social partner, and/or to complete all of the available tasks were common complaints in our exit interviews. However, the value of greater immersion time has to be balanced against increasing risks of negative effects such as simulator sickness. Longitudinal studies across multiple immersion sessions may be an optimal way to evaluate the impact of increased exposure.

The extent of researcher moderation in VR social activities is also an important variable. Our study used a relatively heavy moderation approach, particularly in the first two modules, as the research assistants were heavily involved in initiating introductory conversations. Further, participants were assigned to a random social partner and did not have a selection choice. While a more open-ended social design might be preferable in some ways, it is important to think carefully about these parameters and to evaluate their impact, especially when working with potentially vulnerable populations such as individuals with CI. There is a great research need in this area to determine the optimal means of balancing user autonomy against potential harms (not to mention moderator fatigue) in the service of achieving the social program’s stated goals. Finally, the current study was limited to self-reported metrics for variables such as mood, affective response, and engagement. These survey instruments are valuable and effective research tools, but they may be usefully supplemented in future work with physiological data such as eye tracking, heart rate, and EEG (Electroencephalography) to provide additional information about how older adults experience social VR.

## Supplementary Material

igad031_suppl_Supplementary_Material

## Data Availability

The data sets presented in this study can be found in online repositories. The anonymized quantitative and qualitative data, and R code, are available via the following online OSF link: https://osf.io/4sxfn/.

## References

[CIT0001] Appel, L., Appel, E., Bogler, O., Wiseman, M., Cohen, L., Ein, N., Abrams, H. B., & Campos, J. L. (2020). Older adults with cognitive and/or physical impairments can benefit from immersive virtual reality experiences: A feasibility study. Frontiers in Medicine, 6, 329. doi:10.3389/fmed.2019.00329 32010701 PMC6974513

[CIT0002] Arlati, S., di Santo, S. G., Franchini, F., Mondellini, M., Filiputti, B., Luchi, M., Ratto, F., Ferrigno, G., Sacco, M., & Greci, L. (2021). Acceptance and usability of immersive virtual reality in older adults with objective and subjective cognitive decline. Journal of Alzheimer’s Disease, 80(3), 1025–1038. doi:10.3233/jad-201431 33646164

[CIT0003] Baker, S., Kelly, R. M., Waycott, J., Carrasco, R., Hoang, T., Batchelor, F., Ozanne, E., Dow, B., Warburton, J., & Vetere, F. (2019). Interrogating social virtual reality as a communication medium for older adults. Proceedings of the ACM on Human–Computer Interaction, 3(CSCW), 1–24. doi:10.1145/335925134322658

[CIT0004] Barbeite, F. G., & Weiss, E. M. (2004). Computer self-efficacy and anxiety scales for an Internet sample: Testing measurement equivalence of existing measures and development of new scales. Computers in Human Behavior, 20(1), 1–15. doi:10.1016/S0747-5632(03)00049-9

[CIT0005] Barreda-Ángeles, M., & Hartmann, T. (2022). Psychological benefits of using social virtual reality platforms during the covid-19 pandemic: The role of social and spatial presence. Computers in Human Behavior, 127, 107047. doi:10.1016/j.chb.2021.107047 34629723 PMC8489850

[CIT0006] Benford, S., Greenhalgh, C., Rodden, T., & Pycock, J. (2001). Collaborative virtual environments. Communications of the ACM, 44(7), 79–85. doi:10.1145/379300.379322

[CIT0007] Blackwell, L., Ellison, N., Elliott-Deflo, N., & Schwartz, R. (2019). Harassment in social virtual reality: Challenges for platform governance. Proceedings of the ACM on Human–Computer Interaction, 3(CSCW), 1–25. doi:10.1145/335920234322658

[CIT0008] Bol, N., Høie, N. M., Nguyen, M. H., & Smit, E. S. (2019). Customization in mobile health apps: Explaining effects on physical activity intentions by the need for autonomy. Digital Health, 5, 2055207619888074. doi:10.1177/2055207619888074 31807312 PMC6880050

[CIT0009] Boothby, E. J., Cooney, G., Sandstrom, G. M., & Clark, M. S. (2018). The liking gap in conversations: Do people like us more than we think? Psychological Science, 29(11), 1742–1756. doi:10.1177/0956797618783714 30183512

[CIT0010] Brandt, A., Jensen, M. P., Søberg, M. S., Andersen, S. D., & Sund, T. (2020). Information and communication technology-based assistive technology to compensate for impaired cognition in everyday life: A systematic review. Disability and Rehabilitation: Assistive Technology, 15(7), 810–824. doi:10.1080/17483107.2020.1765032 32407217

[CIT0011] Bradley, M. M., & Lang, P. J. (1994). Measuring emotion: The self-assessment manikin and the semantic differential. Journal of Behavior Therapy and Experimental Psychiatry, 25(1), 49–59. doi:10.1016/0005-7916(94)90063-9 7962581

[CIT0012] Cooke, R. G., Robb, J. C., Young, L. T., & Joffe, R. T. (1996). Well-being and functioning in patients with bipolar disorder assessed using the MOS 20-ITEM short form (SF-20). Journal of Affective Disorders, 39(2), 93–97. doi:10.1016/0165-0327(96)00016-x 8827417

[CIT0013] Cotten, S. R., Anderson, W. A., & McCullough, B. M. (2013). Impact of Internet use on loneliness and contact with others among older adults: A cross-sectional analysis. Journal of Medical Internet Research, 15(2), e39. doi:10.2196/jmir.2306 23448864 PMC3636305

[CIT0014] Cotterell, N., Buffel, T., & Phillipson, C. (2018). Preventing social isolation in older people. Maturitas, 113, 80–84. doi:10.1016/j.maturitas.2018.04.014 29903652

[CIT0015] Czaja, S. J., Boot, W. R., Charness, N., Rogers, W. A., & Sharit, J. (2018). Improving social support for older adults through technology: Findings from the PRISM randomized controlled trial. Gerontologist, 58(3), 467–477. doi:10.1093/geront/gnw249 28201730 PMC5946917

[CIT0016] Czaja, S. J., Charness, N., Fisk, A. D., Hertzog, C., Nair, S. N., Rogers, W. A., & Sharit, J. (2013). Factors predicting the use of technology: Findings from the Center for Research and Education on Aging and Technology Enhancement (CREATE). Psychology and Aging, 28(4), 984–998. doi:10.1037/a0034241 16768579 PMC1524856

[CIT0017] D’Cunha, N. M., Nguyen, D., Naumovski, N., McKune, A. J., Kellett, J., Georgousopoulou, E. N., Frost, J., & Isbel, S. (2019). A mini-review of virtual reality-based interventions to promote well-being for people living with dementia and mild cognitive impairment. Gerontology, 65(4), 430–440. doi:10.1159/000500040 31108489

[CIT0018] Darfler, M., Cruz-Garza, J. G., & Kalantari, S. (2022). An EEG-based investigation of the effect of perceived observation on visual memory in virtual environments. Brain Sciences, 12(2), 269. doi:10.3390/brainsci12020269 35204033 PMC8870655

[CIT0019] Dermody, G., Whitehead, L., Wilson, G., & Glass, C. (2020). The role of virtual reality in improving health outcomes for community-dwelling older adults: Systematic review. Journal of Medical Internet Research, 22(6), e17331. doi:10.2196/17331 32478662 PMC7296414

[CIT0020] Dilanchian, A. T., Andringa, R., & Boot, W. R. (2021). A pilot study exploring age differences in presence, workload, and cybersickness in the experience of immersive virtual reality environments. Frontiers in Virtual Reality, 2, 1–11. doi:10.3389/frvir.2021.736793

[CIT0021] Elo, S., & Kyngäs, H. (2008). The qualitative content analysis process. Journal of Advanced Nursing, 62(1), 107–115. doi:10.1111/j.1365-2648.2007.04569.x 18352969

[CIT0022] Fakoya, O. A., McCorry, N. K., & Donnelly, M. (2020). Loneliness and social isolation interventions for older adults: A scoping review of reviews. BMC Public Health, 20(1), 1–14. doi:10.1186/s12889-020-8251-6 32054474 PMC7020371

[CIT0023] Finstad, K. (2010). The usability metric for user experience. Interacting with Computers, 22(5), 323–327. doi:10.1016/j.intcom.2010.04.004

[CIT0024] Freeman, G., & Maloney, D. (2021). Body, avatar, and me: The presentation and perception of self in social virtual reality. Proceedings of the ACM on Human–Computer Interaction, 4(CSCW3), 1–27. doi:10.1145/3432938

[CIT0025] Freeman, G., Zamanifard, S., Maloney, D., & Adkins, A. (2020). My Body, My Avatar: How People Perceive Their Avatars in Social Virtual Reality. Extended Abstracts of the 2020 CHI Conference on Human Factors in Computing Systems, 1–8. Honolulu HI USA: ACM. doi: 10.1145/3334480.3382923

[CIT0026] Hart, S. G., & Staveland, L. E. (1988). Development of NASA-TLX (Task Load Index): Results of empirical and theoretical research. Advances in Psychology, 52, 139–183. doi:10.1016/S0166-4115(08)62386-9

[CIT0027] Herzog, W., & Boomsma, A. (2009). Small-sample robust estimators of noncentrality-based and incremental model fit. Structural Equation Modeling: A Multidisciplinary Journal, 16(1), 1–27. doi:10.1080/10705510802561279

[CIT0028] Herzog, W., Boomsma, A., & Reinecke, S. (2007). The model-size effect on traditional and modified tests of covariance structures. Structural Equation Modeling: A Multidisciplinary Journal, 14(3), 361–390. doi:10.1080/10705510701301602

[CIT0029] Hill, N. T. M., Mowszowski, L., Naismith, S. L., Chadwick, V. L., Valenzuela, M., & Lampit, A. (2017). Computerized cognitive training in older adults with mild cognitive impairment or dementia: A systematic review and meta-analysis. American Journal of Psychiatry, 174(4), 329–340. doi:10.1176/appi.ajp.2016.16030360 27838936

[CIT0030] Hooper, D., Coughlan, J., & Mullen, M. R. (2008). Structural equation modelling: Guidelines for determining model fit. Electronic Journal of Business Research Methods, 6(1), 53–60. https://academic-publishing.org/index.php/ejbrm/article/view/1224/1187

[CIT0031] Huygelier, H., Schraepen, B., van Ee, R., vanden Abeele, V., & Gillebert, C. R. (2019). Acceptance of immersive head-mounted virtual reality in older adults. Scientific Reports, 9(1), 4519. doi:10.1038/s41598-019-41200-6 30872760 PMC6418153

[CIT0032] Jonas, M., Said, S., Yu, D., Aiello, C., Furlo, N., & Zytko, D. (2019). Towards a taxonomy of social VR application design. Extended Abstracts of the Annual Symposium on Computer–Human Interaction in Play Companion Extended Abstracts (pp. 437–444). Barcelona Spain: Association for Computing Machinery. doi:10.1145/3341215.3356271

[CIT0033] Kalantari, S., & Neo, J. R. J. (2020). Virtual environments for design research: Lessons learned from use of fully immersive virtual reality in interior design research. Journal of Interior Design, 45(3), 27–42. doi:10.1111/joid.12171

[CIT0034] Kalantari, S., Xu, T., Mostafavi, A., Lee, A., Barankevich, R., Boot, W. R., & Czaja, S. J. (2022). Using a nature-based virtual reality environment for improving mood states and cognitive engagement in older adults: A mixed-method feasibility study. Innovation in Aging, 6(3), igac015. doi:10.1093/geroni/igac015 35592668 PMC9113189

[CIT0035] Kennedy, R. S., Lane, N. E., Berbaum, K. S., & Lilienthal, M. G. (1993). Simulator Sickness Questionnaire: An enhanced method for quantifying simulator sickness. The International Journal of Aviation Psychology, 3(3), 203–220. doi:10.1207/s15327108ijap0303_3

[CIT0036] Kolesnichenko, A., McVeigh-Schultz, J., & Isbister, K. (2019). Understanding Emerging Design Practices for Avatar Systems in the Commercial Social VR Ecology. Proceedings of the 2019 on Designing Interactive Systems Conference, 241–252. New York, NY, USA: Association for Computing Machinery. doi: 10.1145/3322276.3322352

[CIT0037] Kontos, E. Z., Emmons, K. M., Puleo, E., & Viswanath, K. (2014). Communication inequalities and public health implications of adult social networking site use in the United States. Journal of Health Communication, 19(2), 210–220. doi:10.1080/10810730.2013.798373 21154095 PMC3073379

[CIT0038] Latikka, R., Rubio-Hernández, R., Lohan, E. S., Rantala, J., Nieto Fernández, F., Laitinen, A., & Oksanen, A. (2021). Older adults’ loneliness, social isolation, and physical information and communication technology in the era of ambient assisted living: A systematic literature review. Journal of Medical Internet Research, 23(12), e28022. doi:10.2196/28022 34967760 PMC8759023

[CIT0039] Lee, L. N., Kim, M. J., & Hwang, W. J. (2019). Potential of augmented reality and virtual reality technologies to promote well-being in older adults. Applied Sciences, 9(17), 3556. doi:10.3390/app9173556

[CIT0040] Li, J., Kong, Y., Röggla, T., de Simone, F., Ananthanarayan, S., de Ridder, H., … Cesar, P. (2019). Measuring and understanding photo sharing experiences in social virtual reality. Proceedings of the 2019 CHI Conference on Human Factors in Computing Systems (pp. 1–14). Glasgow, Scotland, UK: ACM. doi:10.1145/3290605.3300897

[CIT0041] Liu, J., Rozelle, S., Xu, Q., Yu, N., & Zhou, T. (2019). Social engagement and elderly health in China: Evidence from the China health and retirement longitudinal survey (CHARLS). International Journal of Environmental Research and Public Health, 16(2), 278. doi:10.3390/ijerph16020278 30669415 PMC6352065

[CIT0042] Lohr, S. L. (2021). Sampling: Design and analysis. Chapman and Hall/CRC.

[CIT0043] Malcolm, M., Frost, H., & Cowie, J. (2019). Loneliness and social isolation causal association with health-related lifestyle risk in older adults: A systematic review and meta-analysis protocol. Systematic Reviews, 8(1), 1–8. doi:10.1186/s13643-019-0968-x 30732659 PMC6366024

[CIT0044] Maloney, D., & Freeman, G. (2020). Falling asleep together: What makes activities in social virtual reality meaningful to users. Proceedings of the Annual Symposium on Computer–Human Interaction in Play (pp. 510–521). Virtual Event Canada: ACM. doi:10.1145/3410404.3414266

[CIT0045] Maloney, D., Freeman, G., & Robb, A. (2020). It is complicated: Interacting with children in social virtual reality. 2020 IEEE Conference on Virtual Reality and 3D User Interfaces Abstracts and Workshops (VRW) (pp. 343–347). IEEE. doi:10.1109/VRW50115.2020.00075

[CIT0046] McCreery, M. P., Vallett, D. B., & Clark, C. (2015). Social interaction in a virtual environment: Examining socio-spatial interactivity and social presence using behavioral analytics. Computers in Human Behavior, 51, 203–206. doi:10.1016/j.chb.2015.04.044

[CIT0047] McGrath, J. E. (1984). Groups: Interaction and performance (Vol. 14). Englewood Cliffs, NJ: Prentice-Hall.

[CIT0048] McVeigh-Schultz, J., Kolesnichenko, A., & Isbister, K. (2019). Shaping pro-social interaction in VR: An emerging design framework. Proceedings of the 2019 CHI Conference on Human Factors in Computing Systems (pp. 1–12). Glasgow Scotland UK: ACM. doi:10.1145/3290605.3300794

[CIT0049] McVeigh-Schultz, J., Márquez Segura, E., Merrill, N., & Isbister, K. (2018). What’s it mean to “be social” in VR? Mapping the social VR design ecology. Proceedings of the 2018 ACM Conference Companion Publication on Designing Interactive Systems (pp. 289–294). Hong Kong China: ACM. doi:10.1145/3197391.3205451

[CIT0050] Mitzner, T. L., Savla, J., Boot, W. R., Sharit, J., Charness, N., Czaja, S. J., & Rogers, W. A. (2019). Technology adoption by older adults: Findings from the PRISM trial. Gerontologist, 59(1), 34–44. doi:10.1093/geront/gny113 30265294 PMC6326254

[CIT0051] Moustafa, F., & Steed, A. (2018). A longitudinal study of small group interaction in social virtual reality. Proceedings of the 24th ACM Symposium on Virtual Reality Software and Technology (pp. 1–10). Tokyo Japan: ACM. doi:10.1145/3281505.3281527

[CIT0052] Nasreddine, Z. S., Phillips, N. A., Bédirian, V., Charbonneau, S., Whitehead, V., Collin, I., Cummings, J. L., & Chertkow, H. (2005). The Montreal Cognitive Assessment, MoCA: A brief screening tool for mild cognitive impairment. Journal of the American Geriatrics Society, 53(4), 695–699. doi:10.1111/j.1532-5415.2005.53221.x 15817019

[CIT0053] National Academies of Sciences and Medicine, E. (2020). Social isolation and loneliness in older adults: Opportunities for the health care system. National Academies Press. 32510896

[CIT0054] Nicholson, N. R. (2012). A review of social isolation: An important but underassessed condition in older adults. The Journal of Primary Prevention, 33(2), 137–152. doi:10.1007/s10935-012-0271-2 22766606

[CIT0055] Nowak, K. L., & Biocca, F. (2003). The effect of the agency and anthropomorphism on users’ sense of telepresence, copresence, and social presence in virtual environments. Presence: Teleoperators and Virtual Environments, 12(5), 481–494. doi:10.1162/105474603322761289

[CIT0056] Nowak, K. L., & Fox, J. (2018). Avatars and Computer-Mediated Communication: A Review of the Uses and Effects of Virtual Representations. Review of Communication Research, 6, 30–53. doi:10.12840/issn.2255-4165.2018.06.01.015

[CIT0057] Park, J.-H., Liao, Y., Kim, D.-R., Song, S., Lim, J. H., Park, H., Lee, Y., & Park, K. W. (2020). Feasibility and tolerability of a culture-based virtual reality (VR) training program in patients with mild cognitive impairment: A randomized controlled pilot study. International Journal of Environmental Research and Public Health, 17(9), 3030. doi:10.3390/ijerph17093030 32349413 PMC7246563

[CIT0058] Roberts, A. R., de Schutter, B., Franks, K., & Radina, M. E. (2019). Older adults’ experiences with audiovisual virtual reality: Perceived usefulness and other factors influencing technology acceptance. Clinical Gerontologist, 42(1), 27–33. doi:10.1080/07317115.2018.1442380 29505343

[CIT0059] Sen, K., Prybutok, G., & Prybutok, V. (2022). The use of digital technology for social well-being reduces social isolation in older adults: A systematic review. SSM: Population Health, 17, 101020. doi:10.1016/j.ssmph.2021.101020 35024424 PMC8733322

[CIT0060] Siriaraya, P., & Ang, C. S. (2012). Characteristics and usage patterns of older people in a 3D online multi-user virtual environment. Computers in Human Behavior, 28(5), 1873–1882. doi:10.1016/j.chb.2012.05.005

[CIT0061] Siriaraya, P., Ang, C. S., & Bobrowicz, A. (2012). Exploring the potential of virtual worlds in engaging older people and supporting healthy aging. Behavior and Information Technology, 33(3), 283–294. doi:10.1080/0144929x.2012.691552

[CIT0062] Siriaraya, P., & Ang, C. S. (2019). The Social Interaction Experiences of Older People in a 3D Virtual Environment. In S. Sayago (Ed.), Perspectives on Human-Computer Interaction Research with Older People (pp. 101–117). Cham: Springer International Publishing. doi:10.1007/978-3-030-06076-3_7

[CIT0063] Sra, M., Mottelson, A., & Maes, P. (2018). Your place and mine: Designing a shared VR experience for remotely located users. Proceedings of the 2018 Designing Interactive Systems Conference (pp. 85–97). Hong Kong China: ACM. doi:10.1145/3196709.3196788

[CIT0064] Steyer, R., Schwenkmezger, P., Notz, P., & Eid, M. (1997). Der Mehrdimensionale Befindlichkeitsfragebogen MDBF [multidimensional mood questionnaire]. Göttingen, Germany: Hogrefe.

[CIT0065] Suh, A., & Prophet, J. (2018). The state of immersive technology research: A literature analysis. Computers in Human Behavior, 86, 77–90. doi:10.1016/j.chb.2018.04.019

[CIT0066] Syed-Abdul, S., Malwade, S., Nursetyo, A. A., Sood, M., Bhatia, M., Barsasella, D., … Li, Y.-C. J. (2019). Virtual reality among the elderly: A usefulness and acceptance study from Taiwan. BMC Geriatrics, 19(1), 1–10. doi:10.1186/s12877-019-1218-8 31426766 PMC6699111

[CIT0067] Swain, A. J. (1975). Analysis of parametric structures for variance matrices/by Anthony J. Swain [Doctoral dissertation]. Unlversity of Adelaide.

[CIT0068] Thach, K. S., Lederman, R., & Waycott, J. (2020). How older adults respond to the use of virtual reality for enrichment: A systematic review. 32nd Australian Conference on Human–Computer Interaction (pp. 303–313). Sydney NSW Australia: Association for Computing Machinery. doi:10.1145/3441000.3441003

[CIT0069] Vaportzis, E., Giatsi Clausen, M., & Gow, A. J. (2017). Older adults’ perceptions of technology and barriers to interacting with tablet computers: A focus group study. Frontiers in Psychology, 8, 1687. doi:10.3389/fpsyg.2017.01687 29071004 PMC5649151

[CIT0070] Vorderer, P., Wirth, W., Gouveia, F. R., Biocca, F., Saari, T., Jäncke, L., Böcking, S., Schramm, H., Gysbers, A., & Hartmann, T. (2004). MEC Spatial Presence Questionnaire (MECSPQ): Short Documentation and Instructions for Application. Report to the European Community, Project Presence: MEC (IST-2001-37661). https://web.archive.org/web/20180410132355id_/http://academic.csuohio.edu/kneuendorf/frames/MECFull.pdf

[CIT0071] Vroman, K. G., Arthanat, S., & Lysack, C. (2015). “Who over 65 is online?” Older adults’ dispositions toward information communication technology. Computers in Human Behavior, 43, 156–166. doi:10.1016/j.chb.2014.10.018

[CIT0072] Williamson, J., Li, J., Vinayagamoorthy, V., Shamma, D. A., & Cesar, P. (2021). Proxemics and social interactions in an instrumented virtual reality workshop. Proceedings of the 2021 CHI Conference on Human Factors in Computing Systems (pp. 1–13). Yokohama Japan: ACM. doi:10.1145/3411764.3445729

[CIT0073] Wolf, E. J., Harrington, K. M., Clark, S. L., & Miller, M. W. (2013). Sample size requirements for structural equation models: An evaluation of power, bias, and solution propriety. Educational and Psychological Measurement, 73(6), 913–934. doi:10.1177/0013164413495237 PMC4334479 25705052

[CIT0074] World Health Organization. (2021). Reducing social isolation and loneliness among older people. Retrieved April 13, 2023, from Reducing social isolation and loneliness among older people website: https://www.who.int/activities/reducing-social-isolation-and-loneliness-among-older-people

[CIT0075] Wu, B. (2020). Social isolation and loneliness among older adults in the context of COVID-19: A global challenge. Global Health Research and Policy, 5(1), 1–3. doi:10.1186/s41256-020-00154-3 32514427 PMC7272234

[CIT0076] Zamanifard, S., & Freeman, G. (2019). “The togetherness that we crave” experiencing social VR in long distance relationships. Conference Companion Publication of the 2019 on Computer Supported Cooperative Work and Social Computing (pp. 438–442). Austin TX USA: ACM. doi:10.1145/3311957.3359453

