# Supplementary material for: Using Immersive Virtual Reality to Enhance Social Interaction Among Older Adults: A Cross-Site Investigation
Source: Innov Aging. 2023 Apr 13;7(4):igad031. doi: 10.1093/geroni/igad031 (PMC10198775; doi:10.1093/geroni/igad031)
Supplement: igad031_suppl_Supplementary_Material [file igad031_suppl_supplementary_material.docx]

**Online Supplementary Material**

**Description of VR Modules**

Participants’ first experience in the VR environment was a **Training Module** that allowed them to become familiar with the technology and its navigational and interaction controls. The virtual training space consisted of a relatively small room with a pleasant floor-to-ceiling window view (Figure 1a). The room contained distinct interaction areas, highlighted in different colors, where participants could read instructional text and practice completing tasks. The skills required to navigate in different sections of VR program were practiced in this module, such as creating an avatar, moving from place to place at different speeds, interacting with textual elements, and grabbing and moving objects. A moderator (one of the researchers) was also available in the training area to answer questions and provide feedback as needed.

The next area that users encountered was an **Introductions Module** or foyer where they could meet other participants and learn about the social activities that were available in the VR. In the current version of the VR environment there was only one possible activity sequence, but in future work we will add additional branching possibilities. The foyer consisted of an expansive, well-lit room with a large world map in the middle (Figure 1b). Upon entering this virtual space, participants were greeted by a moderator (one of the researchers), who served to introduce two participants to each other and help “break the ice” by initiating conversations. The moderator prompted the two participants to share information about themselves such as where they were from and what they were currently doing in their lives (e.g., if they were still working or retired). The moderator then indicated the large world map and prompted discussion about past experiences with traveling and/or places that the participants might have always wanted to visit. The participants were then encouraged to agree on a location to “visit” together in the virtual experience, selecting from a wide variety of available regions or cities indicated on the map.

After agreeing on a destination, the participants engaged in the selected virtual experience in the **Travel Module**, where they watched 360-degree videos, primarily showing major landmarks and recognizable tourist destinations of the chosen location. The participants were encouraged to discuss their experience and what they were seeing. The moderator/researcher accompanied the participants on this journey and, if needed, helped to prompt conversations about features of the environment. Some landmarks in the videos were indicated by name through text appearing in the virtual space. Due to the nature of the video technology, participants were not able to voluntarily move around in these spaces or control the pace of the videos, but they could turn and look in various directions, and see each other’s virtual avatars in the environment (Figure 1c).

After completing the travel segment, participants next encountered the **Productive Engagement Module.** This consisted of a gallery space, where the participants were asked to work together to complete a memory-puzzle task and a creativity task. The moderator/researcher remained available for assistance if requested but did not actively participate. The gallery contained 32 photos from various recognizable world locations, including 8 photos selected from the specific videos that the participants had experienced in the previous Travel module. First, the participants worked together to identify photos from their selected travel destination, and to discard irrelevant photos. They were not graded for accuracy in this task. After agreeing on a set of photos representing their virtual travels, the participants were then asked to organize the photos into a collage on the walls of the room, using any kind of desired artistic arrangement (Figure 1d–e). Like the other modules described above, these activities were intended to present several dimensions of the McGrath circumplex model; in this case the selection of photographs involved memory and intellectual problem-solving (Type 3) and often resolving diverging viewpoints (Type 5), while the collage arrangement served as a creative psychomotor performance task (Type 8).

The virtual environments for the Training, Introduction, and Productive Engagement modules were developed using the Spatial platform v.6.19 (www.spatial.io). For the Travel module we used pre-made 360-degree videos available in the Alcove VR app v.1.194 (www.alcove.com). Audio connectivity was managed through the Zoom app. In the VR design we paid particular attention to the realism of the avatars. The Spatial platform allowed us to automatically generate 3D avatar images based on an uploaded photo of the participant. For the 360-degree video segments, the researchers built custom avatars for each participant using the Oculus Quest’s native avatar system. This is not as precise as Spatial, but it allowed us to roughly customize the avatar’s hair, face, eyes, skin-tone, and outfit in accordance with each participant’s real-life appearance. Previous literature has indicated that users of social VR environments tend to care a great deal about having representative avatars, particularly in terms of color and hairstyle, as it increases their sense of immersion (Ducheneaut et al. 2009). We determined that it would be too time-consuming and potentially distracting to let participants create their own avatars, so instead we completed this task to provide a reasonable representation of each participant’s real-world appearance.

**Description of Measures**

Proficiency with information technology was assessed in the initial demographic instrument using two scales: the *Computer Self-efficacy Scale* (Barbeite & Weiss, 2004) and the *Mobile Device Proficiency Questionnaire* (Roque & Boot, 2018). The first scale focuses on confidence levels in relation to information technology, while the second evaluates proficiency with using various mobile devices (tablet computers and smartphones). Both use 5-point Likert scales, with higher scores indicating greater proficiency. In the same demographic survey, we asked participants to complete the *Positive and Negative Affect Schedule (PANAS)* (Watson, Clark, & Tellegen, 1988). This scale includes 10 items measuring positive affect (e.g., excited, inspired) and 10 items measuring negative affect (e.g., upset, afraid) on a scale between 10 to 60. Finally, the initial demographic instrument also included the *20-Item Short Form Health Survey (SF-20)* (Cooke et al., 1996), which briefly evaluates aspects of physical and mental health functioning.

Immediately prior to the VR sessions, participants’ cognitive capabilities were evaluated using the *Montreal Cognitive Assessment* (Nasreddine et al., 2005). This instrument does not in itself confirm a diagnosis of cognitive impairment (CI), but it is commonly used as a screening tool to assess cognitive status. The assessment involves several brief written and verbal tasks measuring executive functions, memory, language, and reasoning. In the current study the assessment was used to divide the participants into those with a likely cognitive impairment and those without, using a threshold score of less than 26 on the instrument as likely-impaired. A cutoff score of less than 26 is the most commonly used metric for affirming the presence of mild cognitive impairment (Carson et al., 2018; Milani et al., 2018; Wong et al., 2015). None of the participants in the current study scored below 17 on this instrument, which is the standard cutoff to be regarded as having a moderate or severe cognitive impairment.

The mood states of each participant were assessed immediately before and immediately after the VR session, using the *Multidimensional Mood State Questionnaire* (Steyer et al., 1997). This instrument includes 30 items, each on a 6-point Likert scale, with higher scores indicating more positive mood states. The results are divided into three dimensions, including “good/bad” mood (GB), “calm/nervous” mood (CN), and “awake/tired” mood (AT). The total scores of a participant for each of these three dimensions were calculated separately, for both pre- and post-exposure to the VR environment. Immediately before and immediately after the VR session we also assessed attitudes toward VR technology, using a scale developed by Huygelier and colleagues (2019) for the specific purpose of evaluating the *Acceptance of Head-mounted Virtual Reality in Older Adults*. This instrument includes 18 items, each on a 5-point Likert scale, with higher scores indicating a more positive attitude toward the technology. The item scores were summed to obtain a total VR-attitude measurement pre- and post-exposure. After completing each module, participants also completed a *Self-Assessment Manikin (SAM)*. This is a pictographic scale assessed affective reactions in three dimensions of pleasure (happy/unhappy), arousal (excited/bored) and dominance (in control / not in control) (Bradley & Lang, 1994). SAM was repeated after each module, and the final SAM score on each dimension was averaged.

Participants also completed The *MEC Spatial Questionnaire* (Vorderer et al., 2004) after completing the VR tasks. This instrument was used to measure experiences of presence (“being there”) in the virtual environment. The MEC contains two subscales with 6 items each, including the Possible Actions subscale and the Self Location subscale. In addition, participants were asked to complete the *NASA Task Load Index* (Hart & Staveland, 1988) to evaluate perceptions of effort and frustration. This instrument includes six subscales—mental demand, physical demand, temporal demand, effort, performance, and frustration level—each on a 5-point Likert scale, with higher scores indicating greater perceived task loads. Total scores were calculated by summing the responses across all items. They were also asked to complete the *Simulator Sickness Questionnaire* (Kennedy et al., 1993) to assess experiences of “cybersickness.” This instrument includes 16 items on a 4-point Likert scale, with higher scores indicating greater discomfort. The item responses were summed to get a total cybersickness score for each participant.

The level of participants’ *Engagement in the VR Experience* was also assessed following the completion of the VR tasks using a 3-item, 5-point Likert scale developed by the current researchers, with higher scores indicating greater perceived engagement. The Cronbach’s Alpha of 0.81 for this new instrument indicated that there was good internal consistency in the results. To measure the sense that other participants in the environment were “real,” the participants completed the *Social Presence Scale* (Nowak & Biocca, 2003). This instrument involves responding to 6 questions using “sliders” on a scale of 0–100, with higher scores indicating greater perceived social presence. We also used the *Willingness and Likeliness to Reconnect Scale* (Boothby et al., 2018) to assess positive social outcomes. This instrument includes 8 questions in a 5-point Likert scale format to evaluate interest in continuing a social relationship in the future. Higher scores were understood to indicate a more positive view of the social interaction. Finally, participants completed the 4-item *Usability Metric* (Finstad, 2010) after completing the VR immersion. This instrument asked participants to rate the usability of the VR system on a scale of 0–100, with higher scores indicating a more intuitive and comfortable interface.

**Supplementary Tables**

Table S1. *Engagement by Survey Question (5-Point Likert Scale)*

| **Question** | **Mean** | **SD** | **Min.** | **Max.** | **SE** |
| --- | --- | --- | --- | --- | --- |
| I found today’s experience to be engaging. | 4.7 | 0.7 | 2 | 5 | 0.1 |
| The experience I shared with my virtual partner today was meaningful. | 4.0 | 1.1 | 1 | 5 | 0.2 |
| The experience I shared with my partner today allowed me to express creativity. | 3.9 | 1.3 | 1 | 5 | 0.2 |

Table S2. *Social Presence by Survey Question (Sliders on a 0–100 Scale, Reverse-coded)*

| **Question** | **Mean** | **SD** | **Min.** | **Max.** | **SE** |
| --- | --- | --- | --- | --- | --- |
| To what extent did you feel able to assess your partner’s reactions to what you said? | 33.2 | 27.1 | 0 | 100 | 4.5 |
| To what extent was this like a face-to-face meeting? | 48.4 | 31.8 | 1 | 100 | 5.3 |
| To what extent was this like you were in the same room with your partner? | 33.9 | 28.2 | 0 | 100 | 4.7 |
| To what extent did your partner seem “real”? | 30.6 | 31.4 | 0 | 100 | 5.2 |
| How likely is it that you would choose to use this system of interaction for a meeting in which you wanted to persuade others of something? | 50.0 | 33.3 | 0 | 100 | 5.6 |
| To what extent did you feel you could get to know someone that you met only through this system? | 42.6 | 25.4 | 8 | 98 | 4.2 |

Table S3. *Likeliness to Reconnect by Survey Question (5-Point Likert Scale)*

| **Question** | **Mean** | **SD** | **Min.** | **Max.** | **SE** |
| --- | --- | --- | --- | --- | --- |
| I generally liked the other participant. | 4.3 | 0.8 | 2 | 5 | 0.1 |
| I would be interested in getting to know the other participant better. | 3.9 | 1.0 | 2 | 5 | 0.2 |
| If given the chance, I would like to interact with the other participant again. | 3.9 | 1.1 | 2 | 5 | 0.2 |
| I could see myself becoming friends with the other participant. | 3.6 | 1.2 | 1 | 5 | 0.2 |
| The other participant generally liked me. | 3.6 | 0.7 | 3 | 5 | 0.1 |
| The other participant would be interested in getting to know me better. | 3.4 | 0.7 | 2 | 5 | 0.1 |
| If given the chance, the other participant would like to interact with me again. | 3.6 | 0.8 | 2 | 5 | 0.1 |
| The other participant could see himself/herself becoming friends with me. | 3.3 | 0.9 | 1 | 5 | 0.1 |

Table S4. *Results of the SAM Scale Reported for Each Social VR Module; Mean (SD)*

|  | **Introduction Module** | **Travel Module** | **Productive Engagement Module** | **Average** |
| --- | --- | --- | --- | --- |
| **Happy–Unhappy** | 2.5 (1.7) | 2.6 (1.8) | 1.9 (1.6) | 2.3 (1.4) |
| **Excited–Calm** | 4.4 (2.5) | 4.9 (2.4) | 4.7 (2.8) | 4.7 (2.1) |
| **Controlled–Dominant** | 5.0 (2.0) | 4.9 (1.9) | 5.4 (2.1) | 5.1 (1.5) |
